# Exploring Human–Exoskeleton Interaction Dynamics: An In-Depth Analysis of Knee Flexion–Extension Performance across Varied Robot Assistance–Resistance Configurations †

**DOI:** 10.3390/s24082645

**Published:** 2024-04-21

**Authors:** Denis Mosconi, Yecid Moreno, Adriano Siqueira

**Affiliations:** 1 Industry Department, Federal Institute of São Paulo, Catanduva 15808-305, Brazil; 2 Mechanical Engineering Department, University of São Paulo, São Carlos CEP 13566-590, Brazil; yecidmoreno@usp.br (Y.M.); siqueira@sc.usp.br (A.S.); 3 Center of Engineering Applied to Healthy, São Carlos School of Engineering, University of São Paulo, São Carlos CEP 13566-590, Brazil

**Keywords:** knee orthosis, exoskeleton, robotic therapy

## Abstract

Knee rehabilitation therapy after trauma or neuromotor diseases is fundamental to restore the joint functions as best as possible, exoskeleton robots being an important resource in this context, since they optimize therapy by applying tailored forces to assist or resist movements, contributing to improved patient outcomes and treatment efficiency. One of the points that must be taken into account when using robots in rehabilitation is their interaction with the patient, which must be safe for both and guarantee the effectiveness of the treatment. Therefore, the objective of this study was to assess the interaction between humans and an exoskeleton during the execution of knee flexion–extension movements under various configurations of robot assistance and resistance. The evaluation encompassed considerations of myoelectric activity, muscle recruitment, robot torque, and performed movement. To achieve this, an experimental protocol was implemented, involving an individual wearing the exoskeleton and executing knee flexion–extension motions while seated, with the robot configured in five distinct modes: passive (P), assistance on flexion (FA), assistance on extension (EA), assistance on flexion and extension (CA), and resistance on flexion and extension (CR). Results revealed distinctive patterns of movement and muscle recruitment for each mode, highlighting the complex interplay between human and robot; for example, the largest RMS tracking errors were for the EA mode (13.72 degrees) while the smallest for the CR mode (4.47 degrees), a non-obvious result; in addition, myoelectric activity was demonstrated to be greater for the completely assisted mode than without the robot (the maximum activation levels for the vastus medialis and vastus lateralis muscles were more than double those when the user had assistance from the robot). Tracking errors, muscle activations, and torque values varied across modes, emphasizing the need for careful consideration in configuring exoskeleton assistance and resistance to ensure effective and safe rehabilitation. Understanding these human–robot interactions is essential for developing precise rehabilitation programs, optimizing treatment effectiveness, and enhancing patient safety.

## 1. Introduction

The human knee is a hinge joint located between the posterior surface of the patella, the proximal end of the tibia, and the distal end of the femur. Two articulations governed by ligaments constitute it: the tibiofemoral and the patellofemoral articulations [1]. This makes the knee the biggest and most complex synovial joint in the human body. Its movement set is small, limited to flexion and extension in the sagittal plane. Despite this, the knee is essential for the proper execution of movements related to the lower limbs, such as walking, jumping, swimming, lifting, squatting, and climbing.

The restricted set of movements of the knee, along with the fact that it has not much protection, unlike a ball joint (e.g., the hip), and is regularly subjected to stress from both supporting the body weight and absorbing shock from intermittent impacts, makes the knee very vulnerable to traumatic injury [2]. Thus, trauma or neuromotor diseases (e.g., a stroke) can compromise the knee motor skills, impacting the performance of lower limbs movements and reducing the quality of life of the injured. In these cases, rehabilitation therapy is fundamental to restore the joint functions as best as possible [3].

The rehabilitation program should address the restoration of the full range of motion (ROM), strength, neuromuscular control, and full weight bearing (FWB) [4]. One of the various possible movements that can be used during the therapy is the knee flexion–extension (F&E), an open kinetic chain movement that is useful for strengthening, the restoration of joint stability, the improvement of motor coordination, and the augmentation of the range of motion [5]. In the beginning of the treatment, such exercise can be performed in a seated position with the patient being assisted by the therapist. As an improvement in the clinical picture is noted, the therapist can exert a resistance force to the movement performed by the patient, in order to promote strengthening combined with motor control.

A powerful resource that has been increasingly used in the rehabilitation process are exoskeletons robots: wearable robots that can help in the treatment by including forces to assist or resist movement, just like a therapist. Such devices can contribute to a reduction in the therapist workload, time, and costs of the treatment as well as to promote objective prognoses through the data collected with their sensors [6].

One of the points that must be taken into account when using robots in rehabilitation is their interaction with the patient, which must be safe for both and guarantee the effectiveness of the treatment. Thus, it is a good sense that the human–robot interaction is tested and evaluated before using it in a therapy.

In this context, the human–robot interaction was studied by many researchers who took into account the movement performed, the torques applied by the robot, and the myoelectric activity. For example, Ref. [7] evaluated the interaction between an individual and an active orthosis during lifting and lowering movements, concluding that the muscular activity could be reduced with the orthosis assistance, especially when using configurations where the knee was assisted.

An analysis of the human musculoskeletal and energetic adaptation mechanisms related to the interaction with a unilateral knee orthosis during treadmill walking was carried out by [8]. The researchers identified kinematic adaptations only in the assisted joint; however, the activation of the muscles spanning both knees of both legs was affected in order to promote compensation and ensure gait stability. Similar results were found by [9], who also studied the gait symmetry, torque interaction, and muscular response due to the unilateral assistance provided by an active knee orthosis in healthy subjects.

A study to determine the effect of lower-extremity impairment due to exoskeleton knee joint misalignment during gait was conducted by [10]. Four levels of misalignment were designated to examine knee flexors and extensors. Muscle stress variations were observed, with the vastus lateralis muscle showing the most noticeable variations in applied force. Remarkable variations were also observed in the force level of the rectus femoris, biceps femoris long head, and gastrocnemius muscles. These results indicated that the misalignment should be considered when using exoskeletons applied to knee rehabilitation, because, as noted by [11], the uniqueness of every individual does not offer a one-size-fits-allsolution related to the structural conception of rehabilitation robots.

Muscle activations when using a lower-limb assistive orthosis were also observed by [12,13,14] in their works. Refs. [12,13] observed that despite the reduction in muscle fatigue, changes in the movement pattern appeared. Changes in the range of motion and movement speed were also observed by [15], with the use of two-degree-of-freedom knee orthosis on the gait. Ref. [16] reports that despite the reduction in muscle activities, an increase in the metabolic cost resulting from compensatory movements can be noticed when using an unilateral knee exoskeleton during gait.

The aforementioned research shows the importance of studying the interaction between human and exoskeleton robots for rehabilitation, in order to understand how such an interaction occurs with regard to changes in movement, applied torques, possibility of unwanted force exchange, myoelectric activity levels, and muscle recruiting patterns. By understanding human–robot interactions, one can plan an adequate rehabilitation program and ensure treatment effectiveness and patient safety by developing effective interaction controls. This concept is reinforced by the work [17], which affirms that the physical implications of long-term exposure and use of rehabilitation and assistive robots need to be a future direction in research about human–robot interaction focused on rehabilitation.

The purpose of this work was to evaluate a human–exoskeleton interaction during the performance of knee flexion–extension movements, in a seated position, under different configurations of robot assistance–resistance, in order to verify how myoelectric activities and muscle recruitment patterns occurred, as well as to measure the torque applied by the robot and the changes that can occur in the movement. Therefore, this work seeks to promote a better understanding about the human–robot physical interaction for the aforementioned movement, allowing for an adequate application of the orthosis and a more effective and safe treatment planning.

This study was approved by the Ethics Committee of the Federal University of São Carlos (number 26054813.1.0000.5504).

This paper is an extended version of our paper entitled *Human–exoskeleton interaction during knee flexion–extension under different configurations of robot assistance–resistance*, published in *26th International Conference Series on Climbing and Walking Robots and the Support Technologies for Mobile Machines-CLAWAR 2023* [18].

## 2. Materials and Methods

The human–exoskeleton interaction was evaluated from an experiment conducted with an individual wearing a lower-limb exoskeleton and performing movements of knee flexion and extension (F&E), in a seated position, being sometimes assisted and sometimes resisted by the robot, as detailed in the protocol below.

The subject who participated in the experiment was a healthy man, 30 years old, weighing 64.6 kg, and 1.75 m tall. He was right-handed and during the experiment he only performed the movements with his right leg.

**Figure 1:**
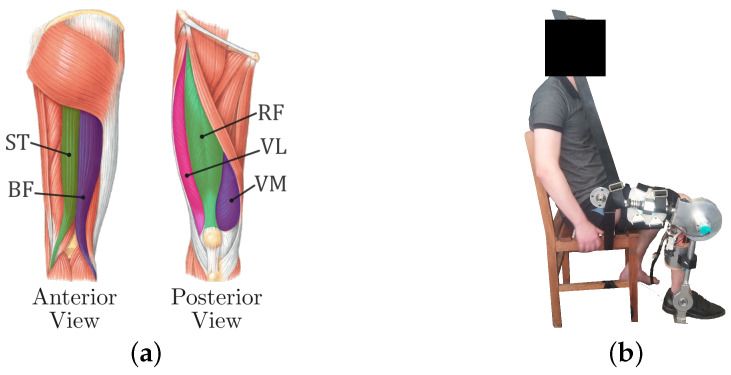
The muscles whose myoelectric signals were measured (**a**) and a subject using the ExoTAO during the experimental procedure (**b**).

The robot used was the ExoTAO, a modular lower-limb exoskeleton developed by [19] whose structure is composed of lightweight tubes connected by six independent free joints, capable of being adjusted to be used by humans with a height between 1.65 and 1.90 m. The modular characteristic of the robot allows it to be applied in the treatment of one to six joints of the patient lower limbs. In this work, only the right knee joint of the robot was used actively, the hip and ankle right joints were used passively, and the left leg of the robot was not used. Figure 1b depicts the subject wearing the ExoTAO during the experiment. The exoskeleton was attached to the human by means of Velcro_®_ straps and a customized shoe, ensuring stability and avoiding joints’ misalignment.

An impedance control law (Equation (1)) was applied to the exoskeleton. In this law, $\tau_{R}$ is the robot torque, $\theta^{d}$ is the knee angular reference to be tracked, θ is the measured knee angle, $K_{R}$ is the robot’s virtual stiffness (which expresses the level of assistance/resistance from the orthosis to the user), $B_{R}$ is the robot virtual damp, and $\dot{\theta }$ is the measured knee angular velocity.

(1) $$\tau_{R}=\left(\theta^{d}-\theta \right)K_{R}-B_{R}\dot{\theta }$$

The experiment protocol consisted of the subject wearing the exoskeleton performing knee flexion–extension in a seated position according to a sinusoidal trajectory with a period of 10 s and an amplitude of $0^{\circ }$ (flexion) to $70^{\circ }$ (extension), for 90 s. Both the reference to be tracked and measured angular trajectories of the knee were displayed to the user on a computer screen. The experiment was divided into six modes:

- **Bare (B):** in this case, the subject performed the movement without wearing the exoskeleton.
- **Passive (P):** performed with the subject wearing the exoskeleton configured in passive mode, with $K_{R}=0$ N/m.
- **Flexion-assisted (FA):** In this case, the exoskeleton was configured to assist in the flexion phase and resist in the extension. The reference of the robot was a fixed value of $0^{\circ }$ and $K_{R}=10$ N/m.
- **Extension-assisted (EA):** In this case, the exoskeleton was configured to assist in the extension phase and resist in the flexion. The reference of the robot was a fixed value of $70^{\circ }$ and $K_{R}=10$ N/m.
- **Completely assisted (CA):** In this case, the exoskeleton was configured to assist both in the flexion and extension phases. The reference to be followed by both the user and the robot were the same and $K_{R}=10$ N/m.
- **Completely resisted (CR):** Here, the exoskeleton resisted the movement performed by the user all the time. To this end, the position reference to be followed by the robot was shifted by 180 degrees in relation to that of the user, and $K_{R}=5$ N/m.

The knee angular position and velocity were measured using Xsens MTw Awinda Wireless Inertial Measurement Units (IMUs) with a work frequency of 100 Hz. The ReRobApp from [20] was used to process the signals from the IMUs. One sensor was placed on the thigh and two on the shank. It was assumed that the knee positions and velocities of both the user and the exoskeleton were the same.

The myoelectric activity of five muscles (Figure 1a) was measured through surface electromyography (sEMG) using a Trigno Wireless EMG System (Delsys Inc., Natic, MA, USA). The muscles considered were the knee flexors biceps femoris (BF) and semitendinosus (ST) and the knee extensors rectus femoris (RM), vastus lateralis (VL), and vastus medialis (VM). The instructions provided by [21] were used for the placement of the electrodes and preparation of the skin (shaving, abrasion with sandpaper, and cleaning with 70% alcohol). The EMG data were normalized to the %MVC that was measured through a maximum voluntary contraction (MVC) procedure were the subject performed an isometric contraction against manual resistance. The EMG data were sampled at 2 kHz on a separate computer using the Delsys EMGworks Software version 4.8.0 and then processed using MATLAB (The MathWorks, Inc.; Natick, MA, USA). First the signal’s moving average (50 ms time window) was subtracted in order to eliminate the DC bias. Then, the signal was rectified and filtered by a second-order Butterworth low-pass filter (cut-off frequency of 2 Hz). Finally, the mean value was extracted and normalized to the MVC mean.

## 3. Results and Discussion

**Figure 2:**
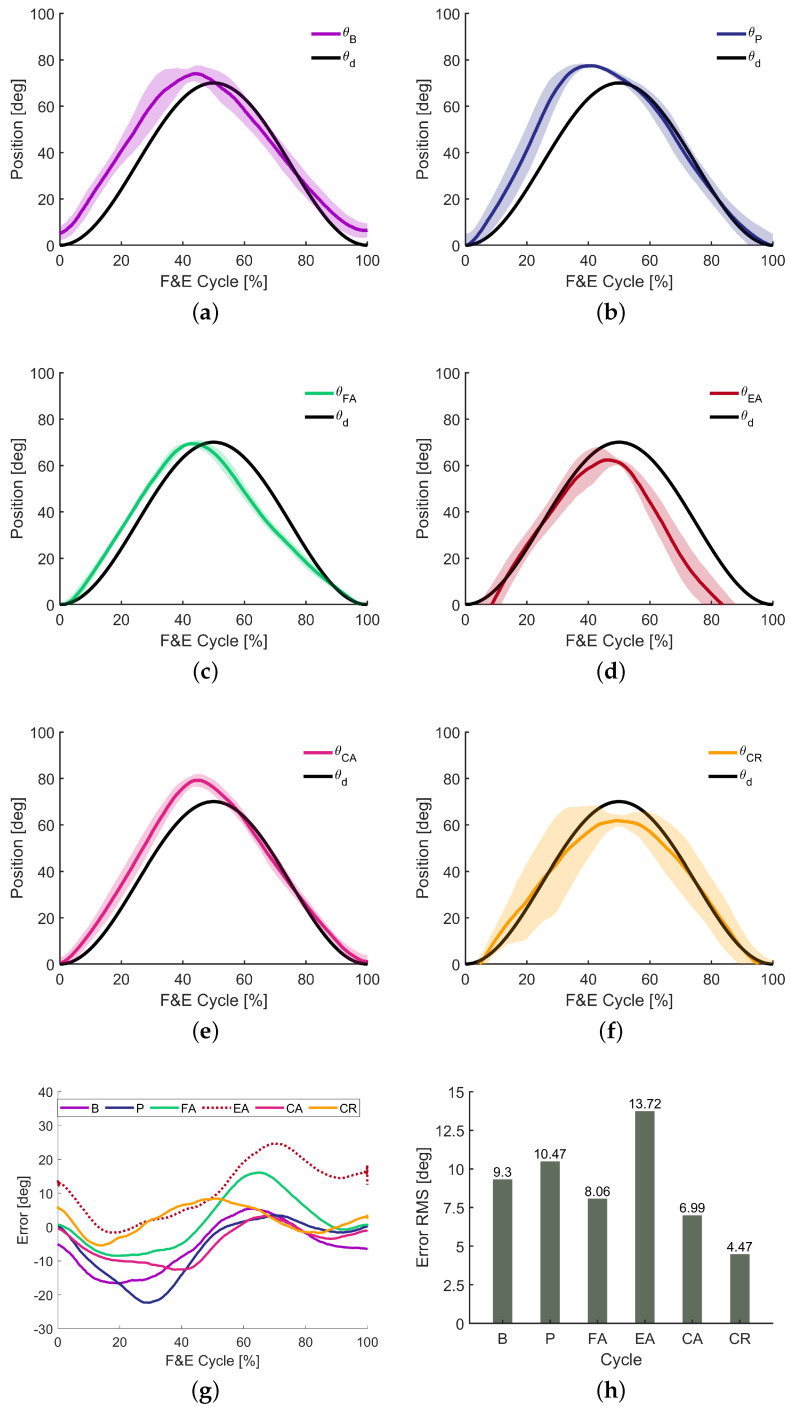
Knee angular position in degrees for the bare $\theta_{B}$ (**a**), passive $\theta_{P}$ (**b**), flexion-assisted $\theta_{FA}$ (**c**), extension-assisted $\theta_{EA}$ (**d**), completely assisted $\theta_{CA}$ (**e**), completely resisted $\theta_{CR}$ (**f**) modes compared with the reference $\theta_{d}$, and the angular (**g**) and RMS (**h**) errors.

The motions performed by the subject during the experiment, as well as the sinusoidal reference to be tracked are presented in Figure 2a–f. The difference between the trajectory reference and the movement performed is the knee angular position error, whose value is depicted in Figure 2g, withe its root mean square (RMS) being presented in Figure 2h.

For the bare mode (Figure 2a), when the individual was not wearing the exoskeleton, in the extension phase, the movement was away from the reference, because the subject started with the knee semi-flexed and developed more velocity in that phase. The maximum extension achieved was greater than that of the reference, in addition to having been reached earlier, both due to the fact that it started out with the knee not fully flexed and the extension speed was higher than desired. In the flexion phase, the knee angular velocity was slightly lower, with the movement ending in a point of semi-flexion of the knee. Thus, despite having developed a sinusoidal trajectory, it is noted that the subject in question tended to develop more strength in extension than in flexion. The movement performed showed reasonable repetitiveness, with greater deviations being identified in the region close to maximum extension, which is appropriate, since in that region, the torque resulting from the weight of the leg is greater, being a resistance for extension and an accelerator for flexion.

In passive mode (Figure 2b), it is noted that in the extension phase, there was an even greater deviation than in bare mode, with the extension peak beyond what was desired. This is due to the fact that in an attempt to overcome the inertia increase from the weight of the robot, the user applied an initial impulse that resulted in an acceleration and speed greater than those necessary for extension, resulting in a movement that was far from what was desired. In the flexion phase, the movement performed was reasonably close to the reference, as this required more refined control from the user, since at that stage, the combined weight of the leg and the robot could result in unwanted acceleration, culminating in high flexion speeds and joint instability.

In the flexion-assisted mode (Figure 2c), the tracking errors for the extension phase were reduced with the subject performing a movement close to the reference; however, there was an increase in tracking error for the flexion phase. In that case, it was possible to verify that the user exerted a flexion velocity beyond what was necessary, which indicated human effort, that is, he did not take advantage of the robot assistance as much as he could. It can be said that the resistance imposed by the robot in the extension helped the user to perform a movement closer to the desired one. However, when no such resistance was encountered during the flexion, the movement moved away from the reference. It is clear that this type of exercise is useful for movement control training, requiring concentration from the user and consequently, contributing to neuroplasticity.

With regard to the extension-assisted mode (Figure 2d), tracking errors for extension were considerably reduced, but, in addition to the maximum desired extension not being applied, a greater deviation was observed in the flexion phase in relation to the other modes. In this case, during extension, the user took better advantage of the robot’s assistance, which helped him to overcome the torque generated by gravity; however, during flexion, in an attempt to overcome the robot’s resistance, as well as the gravity torque, the user applied an exaggerated force, which contributed to a speed greater than that necessary to carry out the movement, which ended at a time before the reference. This result reinforces what was said above about this type of exercise being useful for movement control training.

When completely assisted (Figure 2e), results similar to bare mode were obtained; however, with an improvement in trajectory tracking, especially for the flexion phase. Observing Figure 2e, it is possible to verify that for the CA case, the tracking error was small over time. This is not surprising, as in this case, the user does not experience resistance to movement. However, the tracking errors indicate that some moments exerted more force than necessary to carry out the movement according to the reference trajectory.

For the completely resisted mode, the movement closest to the proposed reference was obtained, with extension and flexion performed very close to the desired trajectory. The biggest tracking error in that case was related to the maximum extension, with a movement of smaller amplitude than expected, which was pertinent, since in the sitting position, maximum extension is more difficult to achieve than maximum flexion, especially in a case where in addition to the resistance of gravity, there is opposition from the robot. Furthermore, it is noted that this movement had low repetitiveness, especially in extension.

Considering the movement results obtained, it can be stated that the robot in assistive mode does not always guarantee perfect reference tracking, unless the controller’s gains are increased, but this practice brings with it the disadvantage of reducing participation of the user, which is important especially for neurorehabilitation. Furthermore, the robot in resistive mode does not always cause deviations in following the trajectory and can even help the user to have better control, especially when the individual tends to carry out movements with acceleration, force, and speed above those necessary. The tracking errors are depicted in Figure 2g for each mode, with their root-mean-square (RMS) values presented in Figure 2h.

**Figure 3:**
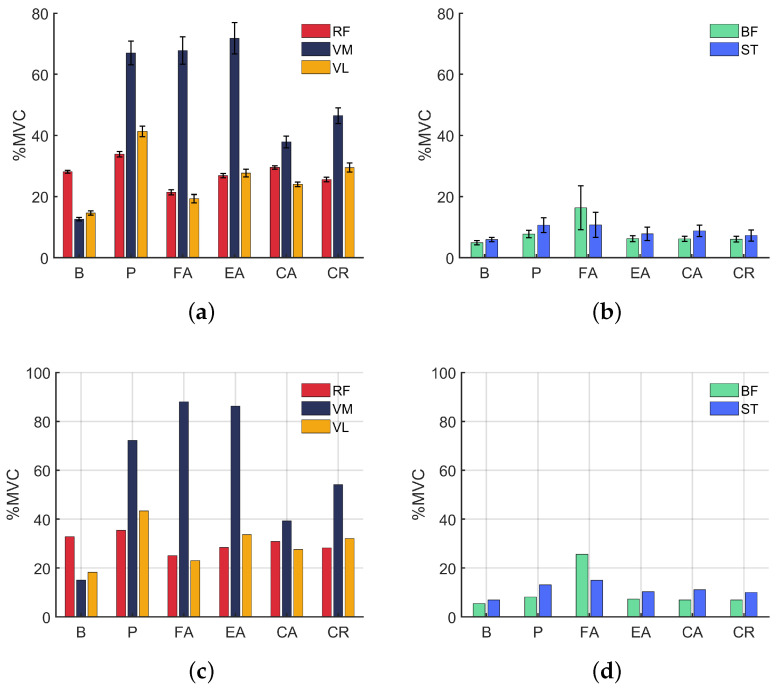
Average value of muscle activations for the extensors (**a**) and flexors (**b**) and maximum muscle activation for the extensors (**c**) and flexors (**d**).

The mean muscle activations in %MVC are depicted in Figure 3a,b. Comparing the activations for the flexors and extensors, it is possible to notice that the flexors were less activated. It is due to the fact that to perform flexion, the user is helped by the force of gravity, while for the extension, such a force acts as a resistance. There was a difference between the recruitment pattern with and without the robot: for the bare mode, the lowest activation belonged to the vastus medialis, while the rectus femoris was the most activated and the vastus lateralis had intermediate levels of activation, whereas in cases with the robot, the vastus medialis was the most activated, while the rectus femoris and vastus lateralis alternated depending on the configuration. Furthermore, regarding the activation of the extensors, it is possible to see that when using the orthosis, the vastus medialis was reasonably requested, in order to ensure the patellar stability through the maintenance of the tibiofemoral alignment.

In passive mode (P), it is noted that there was an increase in extensor activations compared to the bare mode. This is due to the fact that in that case, the user must overcome, in addition to the weight of the leg, the weight of the robot. Furthermore, the initial impulse of the extension contributed to such activation values, a fact corroborated by the maximum activation levels presented in Figure 3c. In the case of the flexors, there was also an increase in the level of activation, as these muscles function as brakes for the extension, which was faster than desired, requiring more braking work from this muscle group.

In the flexion-assisted mode (FA) the extensors acted more than in the bare mode, because the user faced a resistance to perform the extension. Analyzing the muscle activation of the flexors, we can see that in this mode, the biceps femoris (BF) and the semitendinosus (ST) were more activated when compared with the bare mode. The main cause of this is the fact that in the flexion phase of the movement, the angular acceleration was greater, since there was no resistance at that time. This is confirmed by observing Figure 2c, which indicates a greater velocity in flexion than in extension. In this mode, the muscle recruitment pattern was inverted in relation to the bare mode, with the BF acting more than the ST.

For the extension-assisted mode (EA), the extensors were also more activated than in the bare mode due the peak acceleration in the assisted phase. The flexors showed less activation than in the FA mode, indicating that during the flexion phase of the EA mode, the user was more carried away by the robot, a fact proven by the trajectory-following errors in that phase and mode that can be observed in Figure 2d.

In the CA mode, the extensor muscles worked less than in the FA and EA modes, but more than in the bare mode; considering this and the fact that in this condition, tracking errors were reduced (Figure 2e), it can be inferred that in this configuration, the user took better advantage of the robot’s assistance to execute the movement. In all the modes where the subject used the exoskeleton, the recruitment pattern was different from the one observed for the bare mode: with the robot, the VM was the more activated muscle, seeking to ensure the patellar stability, leading us to conclude that the exoskeleton may include some force that may cause an unwanted rotation of the knee.

In the EA and CA cases, it was possible to notice an little increase in the BF activation, while a decrease in the ST activation was perceived, when compared with the bare mode. For these EA and CA modes, the recruitment pattern of ST working more than BF was maintained, as in the bare mode.

For the completely resisted (CR) mode, the vastus medialis activation levels were lower than in the other modes such as P, FA, and EA modes. The rectus femoris was also less activated than in the CA, P, and B modes. The vastus lateralis was only less activated than in the P mode. This difference in activation in relation to the other modes is due to the fact that in this case, the movement is more controlled, without large impulses in extension (a fact that causes a high activation of the extensors for an acceleration as well as a high flexor activation for braking). Furthermore, the maximum reference extension was not reached, so the muscles did not experience maximum concentric contraction, which resulted in an overall movement with lower levels of activation. As for the flexors, little difference was identified in relation to the bare mode, the main reason being the fact that in flexion, the user had the assistance of the force of gravity to complete the movement.

A possible factor that may have contributed to the increase in the activation of the vastus medialis muscle in the modes using the robot is the misalignment of the exoskeleton and knee joint. This consideration is based on the work of [10] which observed an augmentation in muscle activations due to a misalignment between robot and human knee. Therefore, in future experiments, we intend to evaluate the issue of alignment between the joints of the robot and the subject.

**Figure 4:**
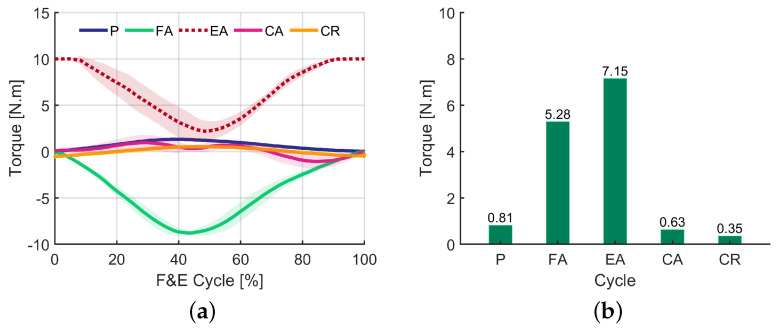
Torque applied by the robot for each mode along with the time (**a**) and RMS (**b**).

The torques applied by the robot over time are presented in Figure 4a. It is important to emphasize that these torques are from a control law, not from predefined patterns from the literature, a resource that will be used in the future for comparison when other experiments will be conducted with more subjects participating. For the passive mode (P), a non-zero torque applied by the robot can be noticed, due to the angular velocity of the knee. It can be seen that when the knee speed was greater, in this case for the extension, the torque applied by the robot was also greater.

Regarding the flexion-assisted (FA) mode, initially the torque was zero, since the movement began with the knee flexed. Then, as the subject performed an extension, the robot increased the torque applied in a temptation to recover the flexed status. After the maximum extension, when the flexion phase started, the torque applied by the exoskeleton decreased until almost zero at the end of the cycle, when the knee was flexed again.

In the extension-assisted (EA) mode, it is possible to notice a torque curve with the same shape of the one obtained for the flexion-assisted mode. However, in this case, the torque initially applied by the robot was greater than zero, as the knee started in the flexion position (the high value of this initial torque is because the reference to be tracked was fixed at 70 degrees, while the subject started the movement with the knee at 0 degrees). As the user performed the extension movement, the torque applied by the robot decreased to almost zero for the maximum extension. Then, during flexion, the robot applied a torque in an attempt to restore the extended position (at that moment the torque was perceived by the user as a resistance to movement).

For the completely assisted mode (CA), little torque was applied by the robot, just in an attempt to correct the reference tracking errors, demonstrating that in general, the user could perform the movement, only needing to be corrected at some points along the way.

For the completely resistive mode, a low torque value was noted, due to the small gain used to control the robot (a high gain could damage the exoskeleton actuator). In this case, the torque started to oppose the extension of the knee, as the user did not actually reach maximum extension, and the robot’s torque approached zero. Finally, at the moment of flexion, the direction of the torque applied by the exoskeleton was reversed; however, it remained numerically low but still sufficient to be perceived by the user as resistance.

The root-mean-square (RMS) values of the torques applied are presented in Figure 4b. The highest value observed is related to assisted extension, while the lowest pertains to a completely resisted movement. The high torque values related to the FA and EA modes are mainly due to the resistance that the robot imposes in each of the modes.

Thus, in general, it can be inferred that the use of an exoskeleton for knee flexion–extension training under different robot assistance–resistance configurations can lead to increased muscle activation, variations in recruitment patterns, and changes in the movement executed.

However, despite the tracking errors observed, the user was able to perform flexion and extension movements within a range approximately close to the desired one, in all tested modes. Thus, it can be stated that the modes studied in this work are useful for training focused on strengthening and developing motor control.

Although the participation of a single subject in the experiment is a limiting factor regarding the generalization of the results, it is plausible to affirm that the use of an exoskeleton robot causes variations in the levels of myoelectric activity, pattern of muscle recruitment, and movement execution. Therefore, this research highlights the complexity of human–robot interaction in rehabilitation settings and underscores the importance of understanding these dynamics for developing precise rehabilitation programs, optimizing treatment effectiveness, and enhancing patient safety.

## 4. Conclusions

In conclusion, this study investigated the human–exoskeleton interaction during knee flexion–extension movements in a seated position under various configurations of robot assistance–resistance. The results demonstrated distinct patterns in knee angular positions, isometry, amplitude, and speed for different modes. Notably, the completely resisted mode showed the closest adherence to the desired trajectory, emphasizing the potential for controlled and targeted training. Muscle activation patterns varied across modes, with the exoskeleton influencing flexor and extensor engagement. A torque analysis revealed the dynamic nature of assistance and resistance applied by the robot throughout the movement. Despite tracking errors, the user successfully executed flexion and extension movements in all tested modes, highlighting the utility of these modes for strength training and motor control development. Overall, this research provides valuable insights into optimizing exoskeleton-assisted rehabilitation programs, enhancing effectiveness, and ensuring patient safety.

For future works, we expect to conduct the experiment with a more diverse group of participants, considering variables such as age, gender, and physical condition, changing the pattern of movement and level of assistance–resistance of the robot. We also intend to conduct a longitudinal study to observe the long-term effects of exoskeleton-assisted rehabilitation on muscle strength, joint function, and overall mobility. A comparative study between traditional rehabilitation methods and exoskeleton-assisted rehabilitation is also intended to elucidate the advantages or potential shortcomings of using robotic assistance in therapy.

## Data Availability

The data presented in this study are available on request from the corresponding author.

## References

[1] Panayiotou Charalambous C. (2022). The Knee Made Easy.

[2] Darrow M. (2002). The Knee Sourcebook.

[3] McGinty G., Irrgang J.J., Pezzullo D. (2000). Biomechanical considerations for rehabilitation of the knee. Clin. Biomech..

[4] Brittberg M. (2020). Lower Extremity Joint Preservation: Techniques for Treating the Hip, Knee, and Ankle.

[5] Shelbourne K.D., Biggs A., Gray T. (2007). Deconditioned Knee: The Effectiveness of a Rehabilitation Program that Restores Normal Knee Motion to Improve Symptoms and Function. N. Am. J. Sport. Phys. Ther..

[6] Wilmart R., Garone E., Innocenti B. (2019). The use of robotics devices in knee rehabilitation: A critical review. Muscle Ligaments Tendons J..

[7] Nesler C., Thomas G., Divekar N., Rouse E.J., Gregg R.D. (2022). Enhancing Voluntary Motion with Modular, Backdrivable, Powered Hip and Knee Orthoses. IEEE Robot. Autom. Lett..

[8] Bacek T., Moltedo M., Serrien B., Langlois K., Vanderborght B., Lefeber D., Rodriguez-Guerrero C. (2022). Human Musculoskeletal and Energetic Adaptations to Unilateral Robotic Knee Gait Assistance. IEEE Trans. Biomed. Eng..

[9] Lora-Millan J.S., Moreno J.C., Rocon E. Assessment of gait symmetry, torque interaction and muscular response due to the unilateral assistance provided by an active knee orthosis in healthy subjects. Proceedings of the 2020 8th IEEE RAS/EMBS International Conference for Biomedical Robotics and Biomechatronics (BioRob).

[10] MajidiRad A., Yihun Y., Hakansson N., Mitchell A. (2022). The Effect of Lower Limb Exoskeleton Alignment on Knee Rehabilitation Efficacy. Healthcare.

[11] Levesque L., Doumit M. (2020). Study of human-machine physical interface for wearable mobility assist devices. Med. Eng. Phys..

[12] Zhu H., Nesler C., Divekar N., Peddinti V., Gregg R.D. (2021). Design Principles for Compact, Backdrivable Actuation in Partial-Assist Powered Knee Orthoses. IEEE/ASME Trans. Mechatron..

[13] Villa-Parra A.C., Lima J., Delisle-Rodriguez D., Vargas-Valencia L., Frizera-Neto A., Bastos T. (2020). Assessment of an Assistive Control Approach Applied in an Active Knee Orthosis Plus Walker for Post-Stroke Gait Rehabilitation. Sensors.

[14] Mobarak R., Tigrini A., Verdini F., Al-Timemy A.H., Fioretti S., Burattini L., Mengarelli A. (2024). A Minimal and Multi-Source Recording Setup for Ankle Joint Kinematics Estimation during Walking Using Only Proximal Information from Lower Limb. IEEE Trans. Neural Syst. Rehabil. Eng..

[15] Fesharaki S.A., Farahmand F., Saeedi H., Raeissadat S.A., Abdollahy E., Ahmadi A., Maroufi N. (2020). The Effects of Knee Orthosis with Two Degrees of Freedom Joint Design on Gait and Sit-to-Stand Task in Patients with Medial Knee Osteoarthritis. Sultan Qaboos Univ. Med. J. [SQUMJ].

[16] Lee D., Kwak E.C., McLain B.J., Kang I., Young A.J. (2020). Effects of Assistance during Early Stance Phase Using a Robotic Knee Orthosis on Energetics, Muscle Activity, and Joint Mechanics during Incline and Decline Walking. IEEE Trans. Neural Syst. Rehabil. Eng..

[17] Mohebbi A. (2020). Human-Robot Interaction in Rehabilitation and Assistance: A Review. Curr. Robot. Rep..

[18] Mosconi D., Moreno Y., Siqueira A., Youssef E.S.E., Tokhi M.O., Silva M.F., Rincon L.M. (2024). Human-Exoskeleton Interaction During Knee Flexion-Extension Under Different Configurations of Robot Assistance-Resistance. Proceedings of the Synergetic Cooperation between Robots and Humans.

[19] dos Santos W.M., Nogueira S.L., de Oliveira G.C., Pena G.G., Siqueira A.A.G. Design and evaluation of a modular lower limb exoskeleton for rehabilitation. Proceedings of the 2017 International Conference on Rehabilitation Robotics (ICORR).

[20] Moreno J.Y., Escalante F.M., Boaventura T., Terra M.H., Siqueira A.A. ReRobApp: A modular and open-source software framework for robotic rehabilitation and human-robot interaction. Proceedings of the 2022 9th IEEE RAS/EMBS International Conference for Biomedical Robotics and Biomechatronics (BioRob).

[21] Biomedical Health and Research Program of the European Union SENIAM-Surface ElectroMyoGraphy for the Non-Invasive Assessment of Muscles. http://www.seniam.org/.

